# Comparison between nab-paclitaxel and solvent-based taxanes as neoadjuvant therapy in breast cancer: a systematic review and meta-analysis

**DOI:** 10.1186/s12885-021-07831-7

**Published:** 2021-02-04

**Authors:** Miao Liu, Siyao Liu, Liu Yang, Shu Wang

**Affiliations:** Breast Center, Peking University People’s Hospital, Beijing, China

**Keywords:** Breast cancer, Neoadjuvant chemotherapy, Paclitaxel, Albumin-bound paclitaxel, Meta-analysis

## Abstract

**Background:**

To compare the efficacy and safety of nanoparticle albumin-bound paclitaxel (nab-paclitaxel) and solvent-based taxanes (sb-taxanes) as neoadjuvant therapy in the treatment of breast cancer.

**Methods:**

We systematically searched the PubMed, Embase, and Cochrane Central Register databases. Randomized controlled trials (RCTs) and cohort studies, published in English, about the comparison between nab-paclitaxel and sb-taxanes as neoadjuvant therapy in patients with breast cancer were searched up to September 2019.

**Results:**

The primary outcome was the proportion of patients with pathological complete response (pCR, defined as ypT0 ypN0 or ypT0/is ypN0). Other main outcomes included long-term survival and adverse events (AEs). Seven studies (five RCTs and two cohorts) and 2949 patients were included. Neoadjuvant nab-paclitaxel improved pCR compared with sb-taxanes (ypT0 ypN0: OR = 1.52, 95%CI: 1.27–1.83, *P* < 0.001; ypT0/is ypN0: OR = 1.40, 95%CI: 1.17–1.68, P < 0.001). The benefits of nab-paclitaxel on pCR were persistent in HER2-negative, hormone receptor (HR)-positive breast cancer (OR = 1.53, 95%CI: 1.07–2.19, *P* = 0.020), triple-negative breast cancer (weekly/every 2 weeks regimen; OR = 2.95, 95%CI: 1.54–5.67, *P* < 0.001), and tumors with Ki-67 > 20% (OR = 1.63, 95%CI: 1.26–2.12, P < 0.001). Patients treated with nab-paclitaxel had better event-free survival (EFS; HR = 0.69, 95%CI: 0.57–0.85, P < 0.001) than with sb-taxanes. There were no differences in most of grade > 3 AEs between nab-paclitaxel and sb-taxanes (all *P* > 0.05), besides of any grade hypersensitivity (OR = 0.29, 95%CI: 0.11–0.72, *P* = 0.008), any grade (OR = 2.10, 95%CI: 1.37–3.23, *P* = 0.001) and grade > 3 (OR = 4.01, 95%CI: 2.51–6.41, *P* < 0.001) neuropathy.

**Conclusion:**

Nab-paclitaxel is effective for the treatment of non-metastatic breast cancer in the neoadjuvant setting. Nab-paclitaxel could improve pCR rate and EFS compared with sb-taxanes and with reasonable toxicities.

**Supplementary Information:**

The online version contains supplementary material available at 10.1186/s12885-021-07831-7.

## Background

Breast cancer is the most common malignancy diagnosed in women worldwide [1, 2]. Neoadjuvant chemotherapy is an established treatment to downstage inoperable or locally advanced cancers into operable cancers and to decrease the tumor size, and to allow breast-conserving surgery [2–4]. In addition, neoadjuvant chemotherapy allows the assessment of the tumor response to systemic treatments before surgery [2]. Neoadjuvant chemotherapy has been shown to be at least equivalent to adjuvant chemotherapy in terms of survival [2]. A pathological complete response (pCR) is the best outcome that can be achieved after neoadjuvant chemotherapy and can predict the risk of recurrence [5]. The Food and Drug Administration (FDA) and European Medicines Agency (EMA) have accepted pCR as an endpoint for accelerated drug approval in high-risk early breast cancer.

Conventional solvent-based (sb) taxanes (including paclitaxel and docetaxel) prevent cell proliferation by stabilizing the microtubules and are among the most widely used chemotherapy agents for breast cancer [6, 7]. In the neoadjuvant setting, a regimen of taxane-, alkylator-, and anthracycline-based chemotherapy is a standard-of-care for potentially operable breast cancer [2]. The addition of sb-taxanes to the alkylator−/anthracycline-based regimen improves the clinical response, pCR rate, disease-free survival (DFS), and overall survival (OS) [8]. However, sb-taxanes contain polyethylated castor oil and ethanol to increase the solubility of taxanes, so premedication and long infusion time are necessary to avoid hypersensitivity reactions [9, 10]. In addition, the cremophor excipient traps paclitaxel in micelles, resulting in nonlinear pharmacokinetics [11, 12].

Nanoparticle albumin-bound paclitaxel (nab-paclitaxel) has been designed to avoid solvents and to achieve a higher delivery rate of paclitaxel to the tumors [13]. Several early clinical trials demonstrated that nab-paclitaxel was more effective than the sb-taxanes in the neoadjuvant setting for early breast cancer. And the treatment effect of nab-paclitaxel for breast cancer in the neoadjuvant setting was demonstrated in a previous meta-analysis in 2017, with a pooled pCR rate of 32%, regardless of subtype [14]. However, most of the included studies were single-arm studies, and subgroup comparison was not made between sb-paclitaxel and nab-paclitaxel. Also, in the previous meta-analysis, only three randomized controlled trials (RCTs) were included and showed that using nab-paclitaxel instead of conventional sb-taxanes could improve the pCR rate, with reasonable toxicities [14]. Since 2017, new comparative clinical studies were reported about nab-paclitaxel versus sb-taxanes, and long-term outcomes have been updated for some previous trials [15–22]. Therefore, a comprehensive assessment of the effects of neoadjuvant nab-paclitaxel versus sb-taxanes in breast cancer treatment is needed. The aim of this meta-analysis was to compare the efficacy and safety of nab-paclitaxel versus sb-taxanes as neoadjuvant therapy in breast cancer, with subgroup analysis and long-term clinical outcomes included.

## Methods

### Study design

This was a systematic review and meta-analysis carried out according to the Preferred Reporting Items for Systematic Reviews and Meta-Analyses (PRISMA) guidelines. The PICO (Patient, Intervention, Comparison, Outcome) process, a mnemonic used in evidence-based medicine [23], was used to searching for relevant articles, followed by screening on the basis of the inclusion and exclusion criteria. Study design, subject characteristics, treatment regimen, efficacy, and safety were extracted. All data were reviewed by two independent investigators according to a pre-specified protocol.

### Search strategy

Two authors (YL and LSY) independently searched public databases, including PubMed, Embase, and Cochrane Central Register of Controlled Trial for available literature published up to September 2019. For the PubMed search, we used the MeSH terms ‘breast neoplasm’, ‘neoadjuvant therapy’, and ‘albumin-bound paclitaxel’, as well as other relevant key words. Supplementary Table S1 presents the full search strategies in detail. Abstracts from the American Society of Clinical Oncology (ASCO), the European Society of Medical Oncology Conference (ESMO), and the San Antonio Breast Cancer Symposium (SABCS) were retrieved using similar search terms. We also performed a manual search of the references from the identified articles. The selection and inclusion of the studies were performed in two stages, i.e., the analysis of titles and abstracts, followed by the full texts. Disagreements were resolved by a third author (LM).

### Eligibility criteria

The studies were considered eligible in the presence of the following characteristics: 1) patients with breast cancer with an indication for taxane-based therapy as neoadjuvant chemotherapy; 2) intervention: nab-paclitaxel (Abraxane™); 3) control: sb-taxane (paclitaxel or docetaxel); 4) study design: RCT or cohort study; 5) number of patients ≥30; and 6) language was limited to English. All articles were screened independently by two authors (YL and LSY). For publications reporting on overlapping patients, duplicated records were removed. For multiple publications reporting the same study over time, the last updated data were used. To avoid publication bias, many meta-analyses also include the abstracts of renowned professional annual meetings like ASCO.

### Data extraction and quality assessment

Data extraction was performed in two stages by two independent authors (YL and LSY). Disagreements were resolved by a third author (WS). Data including names of authors, publication year, study design, epidermal growth factor, and HR status, therapy regimen including dosage, pCR (ypT0 ypN0 or ypT0/is ypN0) rate, objective response rate (ORR) before surgery, event-free survival (EFS; event defined as disease progression during neoadjuvant treatment, recurrence, or death), OS (defined as the time from randomization to the date of death in clinical trials or duration of survival after diseases is diagnosed/treated in observational studies), and adverse events (AEs) were extracted using a structured data collection form. ORs of the primary outcome were collected if reported by the observational studies.

Quality assessment was evaluated according to the Cochrane risk bias tool for RCTs [24] and the Newcastle-Ottawa scale (NOS) [25] for observational studies. The Grading of recommendations, assessment, development, and evaluations (GRADE) approach was used to report the confidence of the estimates [26].

### Statistical analysis

Odds ratio (OR) or hazard ratio (HR) and corresponding 95% confidence intervals (CIs) were used to compare the outcomes, namely, post-neoadjuvant chemotherapy pCR, ORR, EFS, OS, and AEs. For studies reporting pCR in different definitions, ypT0 ypN0 was used over ypT0/is ypN0 when reported. Statistical heterogeneity among studies was calculated by Cochran’s Q test and the I^2^ index (> 50% or *P* < 0.10 indicate high heterogeneity) [27]. The random-effect model was used when high statistical heterogeneity was present among studies; otherwise, the fixed-effect model was applied [28]. In addition, the random-effect model was used as a sensitivity analysis for the justification of potential clinical or methodological heterogeneity. The consistency and quality of the results were assessed by sensitivity analysis. The potential publication bias was evaluated using a funnel plot and Egger’s test [29]. *P*-values < 0.05 were considered statistically significant. All analyses were performed using the STATA SE 14.0 package (StataCorp, College Station, Texas, USA). The analyses of the pathological complete response by different definitions for neoadjuvant therapy and the subgroup analyses of pathological complete response (pCR) for neoadjuvant nab-paclitaxel versus sb-taxanes in the treatment of breast cancer according to RCTs/non-RCTs, sb-taxane comparator, sb-paclitaxel, or sb-docetaxel, and HER2 and HR status were preplanned. All other analyses, including subgroups (TNBC, Ki-67, and different regimens) and secondary outcomes (ORR, OS, and EFS), were post hoc and data-driven.

## Results

### Study retrieval and selection

**Fig. 1:**
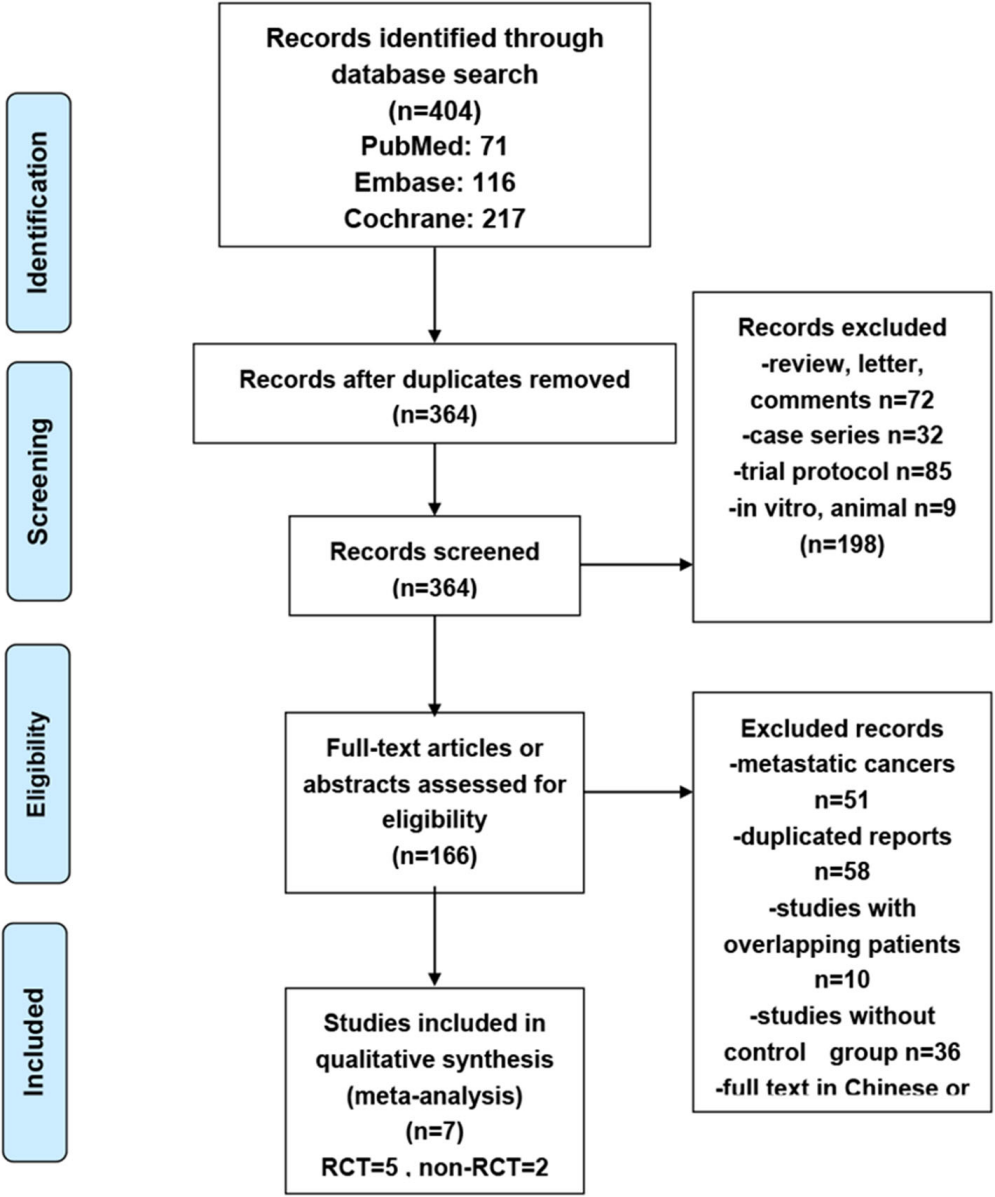
Flowchart of the search process. RCT, randomized controlled trial

Fig. 1 presents the selection flowchart. The initial screening identified 406 papers that met the search parameters. After removing duplicates (*n* = 40 papers) and excluding papers based on article type/study design (*n* = 198 papers) and eligibility criteria (*n* = 159 papers), seven studies remained, including five RCTs [16, 17, 19–22] and two non-RCTs [15, 18] (Table 1). The number of patients per study ranged from 30 to 1206 in RCTs (total, *n* = 2667 patients) and from 120 to 162 in non-RCTs (total, *n* = 282 patients), with a total of 2949 patients across all studies. The age of the included patients ranged from 25 to 79 years. Human epidermal growth factor receptor 2 (HER2) status was reported by all seven studies. Sb-paclitaxel served as the comparator for nab-paclitaxel in five studies and sb-docetaxel in two. Five studies reported pCR by the definition of ypT0 ypN0, and five reported by the definition of ypT0/is ypN0 (Table 1). Untch et al., 2016 [20] and Gianni et al., 2018 [16] were included for pCR primary outcome, and long-term outcomes were extracted from Untch et al. 2019 [21] and Gianni et al., 2019 [30] for analyses. The median follow-up time from Untch et al. 2019 [21] was 49.6 months (range: 0.5–64 months). In the two non-randomized studies, Huang et al. 2015 [15] reported the unadjusted ORs, while Xie et al. 2019 [18] did not report any OR. The disease characteristics and doses of taxanes varied across studies, and the details are provided in Table 1.

**Table 1:** Literature search and study characteristics

Authoryear	n	Trial type	Source	Study phase	Receptor status	Taxane dosage	Neoadjuvant Regimens	n	Age	Stage	pCR definition
Gianni 2018 [16]	695	RCT	Full text	Phase 3	HER2-	125 mg/m^2^ week 1,2,3, q4w*4	nab-p → AC/EC/FEC	346	50 (25–79)	II - III	ypT0/is ypN0
						90 mg/m^2^ week 1,2,3, q4w*4	sb-p → AC/EC/FEC	349	50 (25–79)		
Kuwayama 2018 [17]	152	RCT	Full text	Phase 2	HER2-	100 mg/m^2^ d1,8,15, q4w*4	nab-p → FEC	75	49 (32–73)	I–III	ypT0 ypN0 ypT0/is ypN0
						75 mg/m^2^ q3w*4	docetaxel→FEC	77	51 (25–68)		
Moebus 2018 [22]	598	RCT	Abstract	Phase 3	HER2+; TNBC; luminal B-like;luminal A-like:	330 mg/m^2^ q2w*3	nab-p + EC	298	49 (20–69)	NA	ypT0 ypN0
						60–100 mg/m^2^ q2w*4	docetaxel + EC	300			
Patel 2019 [19]	30	RCT	Full text	Phase 2	HER2+	80 mg/m^2^ qw*12	T-DM1 + L → T-DM1 + L + nab-p	14	53.1 (27.8–69.7)	II or III	ypT0 ypN0
						80 mg/m^2^ qw*12	TP → TP + sb-p	16	57.2 (39.6–74.9)		
Untch 2017 [20, 21]	1206	RCT	Full text	Phase 3	HER2+/−; HR+/−	150 or 125 mg/m^2^ d1,8,15, q3w*4	nab-p → EC	606	49 (43–57)	cT2 to cT4a-d or cT1c with cN/HR−/HER2+/Ki67 > 20%	ypT0 ypN0 ypT0/is ypN0 ypT0/is ypN0/+ ypTany ypN0
						80 mg/m^2^ d1,8,15, q3w*4	sb-p → EC	600	48 (41–56)		
Xie 2019 [18]	162	non-RCT	Full text	NA	HER2+/−; ER and/or PR+, HR-	260 mg/m^2^ q2w*4	EC → nab-p	83	47.8 ± 11.2	cT1–4, cN1–3	ypT0 ypN0; ypT0/is ypN0
						175 mg/m^2^ q2w*4	EC → sb-p	79	52.1 ± 10.4		
Huang 2015 [15]	120	non-RCT	Full text	Phase 2	HER2+/−; HR+/−	125 mg/m^2^ qw*12	nab-p + Cb	30	49 (29–66)	I-III	ypT0/is ypN0; ypT0/is
						80 mg/m^2^ qw*12	paclitaxel + Cb	90	47.5 (24–71)		

Abbreviations: *AC* doxorubicin/cyclophosphamide; *Cb* carboplatin; *cN* clinically assessed axillary node stage; *cT* clinically assessed tumor stage; *EC* epirubicin/cyclophosphamide; *ER* estrogen receptor; *FEC* fluorouracil/epirubicin/cyclophosphamide; *HER2* human epidermal growth factor receptor 2; *HR* hormone receptor; *nab-P* nanoparticle albumin-bound paclitaxel; *P* pertuzumab; *pCR* pathologic complete response; *PR* progesterone receptor;q2w, every 2 weeks; q3w, every 3 weeks; q4w, every 4 weeks; *qw* once weekly; *RCT* randomized controlled trial; *sb-p* solvent-based paclitaxel; *T* trastuzumab; *T-DM1* ado-trastuzumab emtansine; *TNBC* triple-negative breast cancer

### Comparison of efficacy based on pCR

**Fig. 2:**
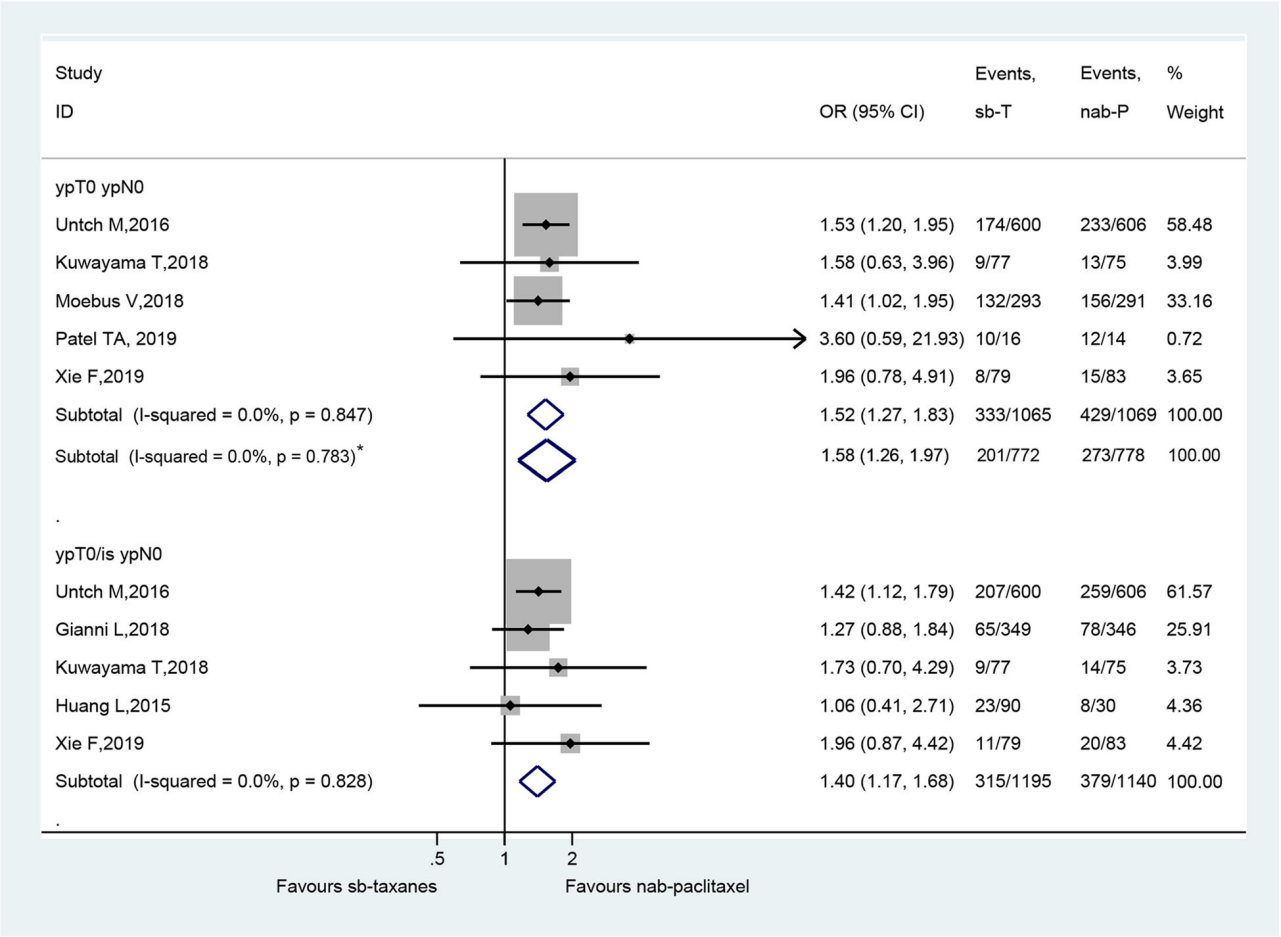
Forest plot of the pathological complete response by different definitions for neoadjuvant nab-paclitaxel versus sb-taxanes in the treatment of breast cancer. Grey boxes demonstrate the effect size of each study, and their size is proportional to the weight given to each study. The whiskers bilateral to each grey box represent the 95% confidence interval (CI) of each study’s effect size. A fixed model was used for odds ratio (OR) calculation. *, sub-total OR when excluding Moebus 2018 [22]

Fig. 2 presents the forest plot of the pCR by different definitions [15–22]. In studies reporting pCR by ypT0 ypN0 (*n* = 5 studies), the pooled proportion of patients with pCR was 40.1% (429/1069) for nab-paclitaxel and 31.3% (333/1065) for sb-taxanes. In studies reporting pCR by ypT0/is ypN0 (n = 5 studies), the pooled proportion of patients with pCR was 33.2% (379/1140) for nab-paclitaxel and 26.4% (315/1195) for sb-taxanes. The pCR rate was significantly higher in patients treated with neoadjuvant nab-paclitaxel than those with sb-taxanes regardless of the definition used (ypT0 ypN0: OR = 1.52, 95%CI: 1.27–1.83, *P* < 0.001; ypT0/is ypN0: OR = 1.40, 95%CI: 1.17–1.68, *P* < 0.001). Regarding the study by Moebus et al. [22], due to differences in both the drug used and the dosing schedule, we also showed subtotal OR (ypT0 ypN0: OR = 1.58, 95%CI: 1.26–1.97) by removing this study, and the conclusion of the meta-analysis did not change (Figs. 2).

### Comparison of efficacy in subgroups

**Fig. 3:**
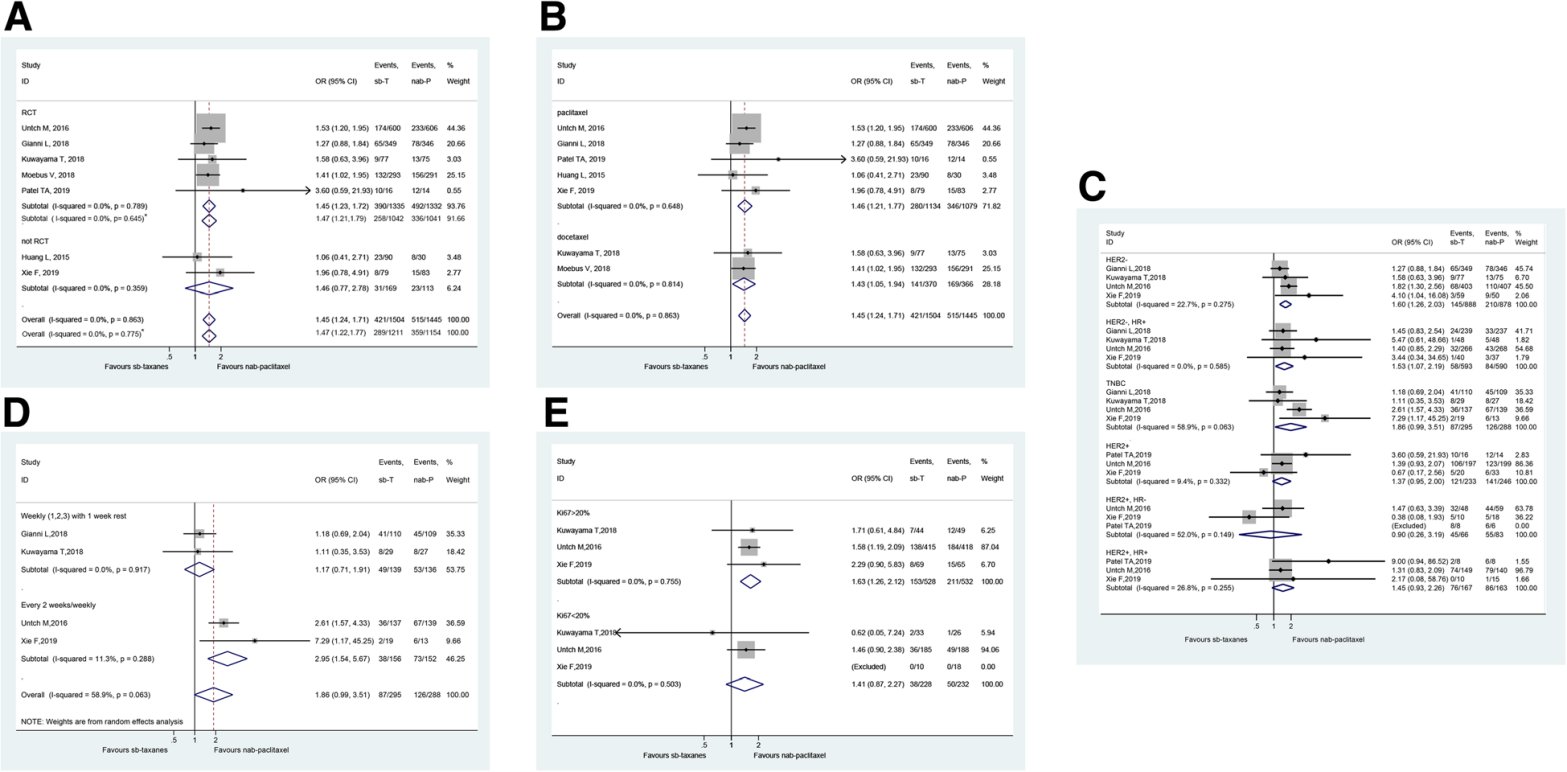
Forest plot of the subgroup analyses of pathological complete response (pCR) for neoadjuvant nab-paclitaxel versus sb-taxanes in the treatment of breast cancer. **a** RCTs and non-RCTs. *, sub-total OR when excluding Moebus 2018 [22]. **b** Sb-taxane comparator, sb-paclitaxel or sb-docetaxel. **c** HER2 and HR status. **d** TNBC. **e** Ki-67. Grey boxes demonstrate the effect size of each study, and their size is proportional to the weight given to each study. The whiskers bilateral to each grey box represent the 95% confidence interval (CI) of each study’s effect size. A random-effects model was used for odds ratio (OR) calculation when high heterogeneity (I^2^ > 50% or *P* < 0.10) was present among studies; otherwise, a fixed-effects model was applied. Abbreviations: nab-P, nanoparticle albumin-bound paclitaxel; sb-T, solvent-based taxanes; RCT, randomized controlled trials; HER2, human epidermal growth factor receptor 2; HR, hormone receptor; TNBC, triple-negative breast cancer

The pCR rate was significantly higher with nab-paclitaxel than sb-taxanes in RCTs (*n* = 5 studies) (pooled pCR: 36.9% vs. 29.2%; OR = 1.45, 95%CI: 1.23–1.72, P < 0.001), but not in non-RCTs (*n* = 2 studies) (pooled pCR: 20.4% vs. 18.3%; OR = 1.46, 95%CI: 0.77–2.78, *P* = 0.177) (Fig. 3a). Removing the study by Moebus et al. [22], the subtotal OR of RCTs was 1.47 (95%CI: 1.21–1.79). The benefit of nab-paclitaxel over sb-taxanes on pCR were also consistent across different comparators (sb-paclitaxel, n = 5 studies or sb-docetaxel, n = 2 studies) (Fig. 3b).

For subtype analysis, pCR benefits of nab-paclitaxel over sb-taxanes were observed in HER2-negative breast cancer (*n* = 4 studies) (pooled pCR: 23.9% vs. 16.3%; OR = 1.60, 95%CI: 1.26–2.03, *P* < 0.001), HER2-negative HR-positive breast cancer (*n* = 4 studies) (pooled pCR: 14.2% vs. 9.8%; OR = 1.53, 95%CI: 1.07–2.19, *P* = 0.020), but not in HER2-positive breast cancer (*n* = 3 studies) (pooled pCR: 57.3% vs. 51.9%; OR = 1.37, 95%CI: 0.95–2.00, *P* = 0.096), HER2-positive HR-negative breast cancer (n = 3 studies) (pooled pCR: 66.3% vs. 68.2%; OR = 0.90, 95%CI: 0.26–3.19, *P* = 0.083), and HER2-positive HR-positive breast cancer (n = 3 studies) (pooled pCR: 52.8% vs. 45.5%; OR = 1.45, 95%CI: 0.93–2.26, *P* = 0.104) (Fig. 3c). For triple-negative breast cancer (TNBC) (n = 4 studies), a trend of improved pCR was observed for nab-paclitaxel versus sb-taxanes (43.8% vs. 29.5%; OR = 1.86, 95%CI: 0.99–3.51, *P* = 0.063), and the strong benefits of nab-paclitaxel over sb-taxanes were observed in TNBC treated with 150/125 mg/m^2^ nab-paclitaxel in day 1, 8, 15 for four 3-week cycles/260 mg/m^2^ four two-weekly cycles regimen (*n* = 2 studies) (pooled pCR: 48.0% vs. 24.4%; OR = 2.95, 95%CI: 1.54–5.67, *P* < 0.001) but not with the 90/100 mg/m^2^ nab-paclitaxel in weeks 1, 2, and 3, followed by a 1-week rest regimen (n = 2 studies) (pooled pCR: 39.0% vs. 35.3%; OR = 1.17, 95%CI: 0.71–1.91, *P* = 0.534) (Fig. 3c and d).

For Ki-67 status, the benefits of nab-paclitaxel over sb-taxanes were observed in tumors with Ki-67 > 20% (*n* = 3 studies) (pooled pCR: 39.7% vs. 29.0%; OR = 1.63, 95%CI: 1.26–2.12, P < 0.001), but not in tumors with Ki-67 < 20% (n = 3 studies) (pooled pCR: 21.6% vs. 16.7%; OR = 1.41, 95%CI: 0.87–2.27, *P* = 0.148) (Fig. 3e).

### Comparison of efficacy based on ORR

**Fig. 4:**
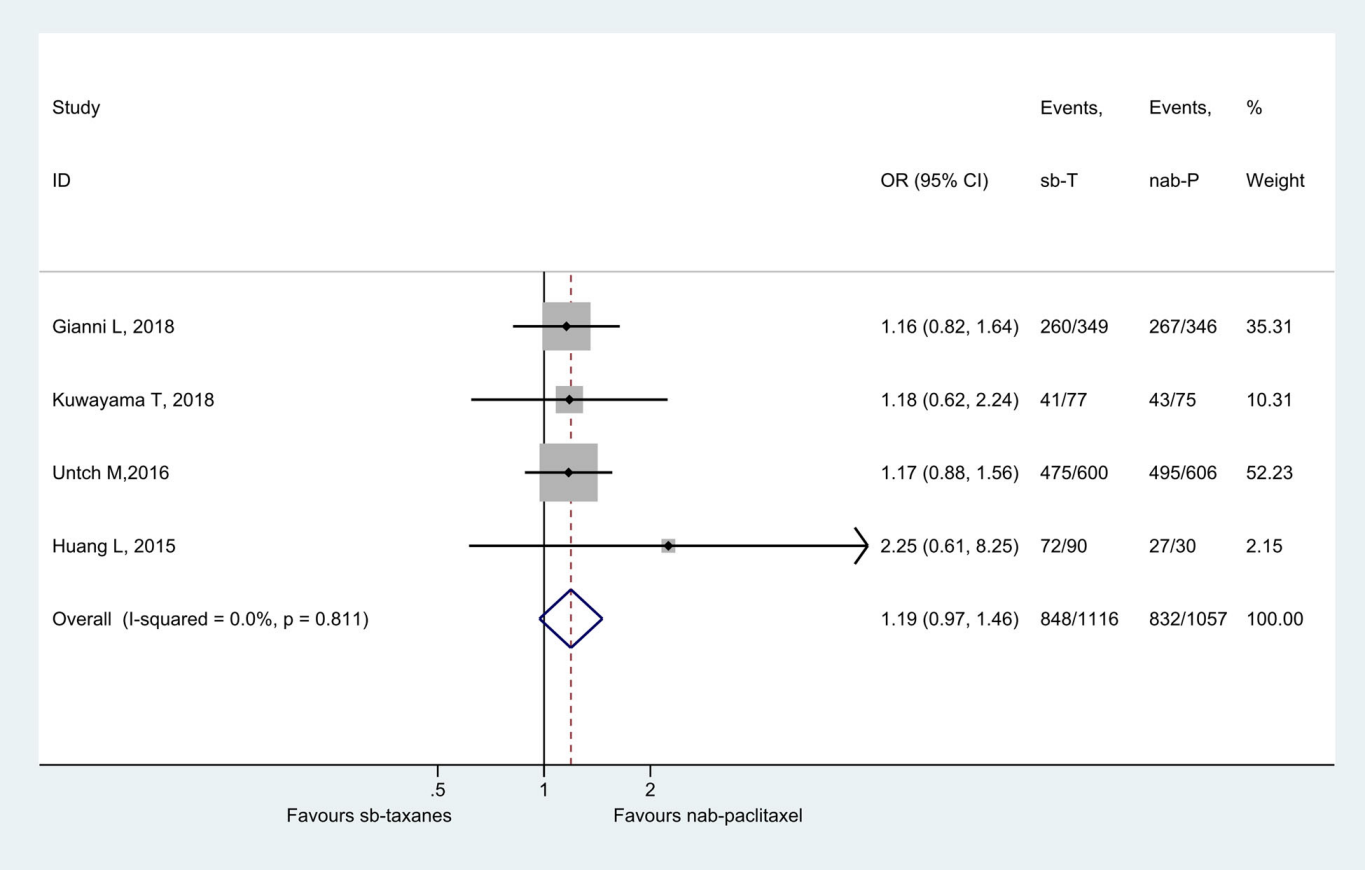
Forest plot of objective response rate for neoadjuvant nab-paclitaxel versus sb-taxanes in the treatment of breast cancer. Grey boxes demonstrate the effect size of each study, and their size is proportional to the weight given to each study. The whiskers bilateral to each grey box represent the 95% confidence interval (CI) of each study’s effect size. A fixed model was used for odds ratio (OR) calculation. Abbreviations: nab-P, nanoparticle albumin-bound paclitaxel; sb-T, solvent-based taxanes

Among the seven studies, four (three RCTs, 2173 patients) had available data for ORR meta-analysis [15–17, 20, 21, 31]. Patients treated with nab-paclitaxel for breast cancer tended to have higher ORR than patients treated with sb-taxanes (n = 3 studies) (I^2^ = 0.0%; OR = 1.19, 95%CI: 0.97–1.46, *P* = 0.094) (Fig. 4).

### Comparison of efficacy based on EFS and OS

Among the seven studies, two RCTs (1901 patients) had available survival data for meta-analysis [16, 20, 21]. Nab-paclitaxel had an EFS benefit over sb-paclitaxel (I^2^ = 48.5%; HR = 0.69, 95%CI: 0.57–0.85, *P* < 0.001) (Figure S1A), but there was no statistically significant difference in OS (I^2^ = 0.0%; OR = 0.79, 95%CI: 0.60–1.04, *P* = 0.092) (Figure S1B).

### Safety profile

The results of all AEs and grade > 3 AEs are listed in Table 2. There were no differences between nab-paclitaxel and sb-taxanes in terms of any-grade increased liver enzymes and liver function abnormalities, rash, arthralgia, alopecia, nausea, and vomiting (all *P* > 0.05). Treatment with nab-paclitaxel was associated with increased occurrence of any-grade neuropathy, fatigue, diarrhea/constipation, neutropenia, myalgia, and anemia (all *P* < 0.05) and reduced occurrence of any-grade hypersensitivity (OR = 0.29, 95%CI: 0.11–0.72, *P* = 0.008), compared with sb-taxanes. The occurrence of grade > 3 AEs was, in general, similar between the two treatment regimens, excluding neuropathy. The occurrence of grade > 3 neuropathy was higher in patients treated with nab-paclitaxel (OR = 4.01, 95%CI: 2.51–6.41, *P* < 0.001) than with sb-taxanes.

**Table 2:** Adverse events

			nab-P		sb-T					
		n	Event	Total	Event	Total	OR (95%CI)	P for OR	I^2^	P for I^2^
Neuropathy	Any grade	6	844	1127	676	1182	2.10 (1.37, 3.23)	0.001	71.6	0.003
	Grade ≥ 3	4	87	1083	23	1076	4.01 (2.51, 6.41)	< 0.001	0.0	0.458
Increased ALT	Any grade	2	364	942	379	936	0.91 (0.74, 1.12)	0.356	42.3	0.188
	Grade ≥ 3	2	16	942	17	936	0.93 (0.47, 1.86)	0.846	61.0	0.109
Increased AST	Any grade	2	250	942	235	936	1.09 (0.87, 1.36)	0.451	0.9	0.315
	Grade ≥ 3	2	6	942	6	936	0.99 (0.33, 2.96)	0.991	32.5	0.224
Liver function abnormalities	Any grade	1	11	14	9	16	2.85 (0.57, 14.33)	0.203	NA	NA
	Grade ≥ 3	1	2	14	0	16	6.60 (0.29, 150.07)	0.236	NA	NA
Fatigue	Any grade	4	670	1030	622	1029	1.27 (1.04, 1.55)	0.018	0.0	0.495
	Grade ≥ 3	4	39	1030	29	1029	1.35 (0.83, 2.20)	0.225	0.0	0.622
Diarrhea/constipation	Any grade	5	429	1060	420	1119	1.21 (1.01, 1.45)	0.010	0.0	0.638
	Grade ≥ 3	4	28	1046	22	1103	1.35 (0.77, 2.36)	0.297	0.0	0.713
Rash	Any grade	3	61	381	71	441	1.15 (0.78, 1.69)	0.484	0.0	0.634
	Grade ≥ 3	2	1	367	3	425	1.08 (0.02, 67.43)	0.970	71.9	0.059
Neutropenia	Any grade	5	765	1060	748	1119	1.53 (1.24, 1.89)	< 0.001	0.9	0.401
	Grade ≥ 3	4	528	1046	520	1103	1.33 (0.74, 2.39)	0.333	81.2	0.001
Leucopenia	Any grade	4	731	1046	766	1103	1.22 (0.93, 1.58)	0.146	0.0	0.831
	Grade ≥ 3	4	347	1046	353	1103	1.29 (0.73, 2.320)	0.380	75.9	0.006
Hypersensitivity	Any grade	1	6	337	20	335	0.29 (0.11, 0.72)	0.008	NA	NA
	Grade ≥ 3	1	1	337	2	335	0.50 (0.04, 5.49)	0.567	NA	NA
Myalgia	Any grade	2	211	679	176	678	1.29 (1.02, 1.63)	0.036	0.0	0.334
	Grade ≥ 3	1	2	679	0	678	4.98 (0.24, 104.02)	0.3	NA	NA
Arthralgia	Any grade	2	249	679	239	678	1.06 (0.85, 1.33)	0.582	10.7	0.29
	Grade ≥ 3	1	5	679	1	678	5.0 (0.58, 42.92)	0.140	NA	NA
Alopecia	Any grade	2	608	679	627	678	0.50 (0.12, 2.12)	0.347	90.1	0.001
	Grade ≥ 3	2	0	679	0	678	NA	NA	NA	NA
Anemia	Any grade	2	588	635	597	691	1.86 (1.28, 2.71)	0.001	23.3	0.254
	Grade ≥ 3	2	18	635	7	691	3.89 (1.55, 9.72)	< 0.001	0.0	0.547
Nausea	Any grade	5	688	1060	738	1119	1.02 (0.85, 1.22)	0.869	0.0	0.463
	Grade ≥ 3	3	30	1046	28	1103	1.12 (0.67, 1.89)	0.659	9.0	0.333
Vomiting	Any grade	4	266	1046	299	1103	1.13 (0.92, 1.39)	0.253	9.5	0.345
	Grade ≥ 3	3	23	1046	19	1103	1.30 (0.70, 2.39)	0.404	46.2	0.156

Abbreviations: *ALT* alanine aminotransferase; *AST* aspartare aminotransferase; *CI* confidence interval; *NA* not applicable; *nab-P* nanoparticle albumin-bound paclitaxel; *OR* odds ratio; *sb-T* solvent-based taxanes

### Quality assessment

The results showed that the quality of the included studies ranged from moderate to high (Supplementary Figure S2 and Supplementary Table 2). For assessments of bias, five RCTs had low or unclear risk. Gianni et al. [16] and Untch et al. [20] had low risk for all bias. Kuwayama et al. [17] had unsure risk of bias for randomization, allocation concealment and other bias (without registration), Patel et al. [19] had an unsure risk of bias for randomization and allocation concealment, while Moebus et al. [22] had an unclear risk of bias for allocation concealment (Supplementary Figure S2). Both the NOS score of two observational studies was 9, indicating that the included studies were with high quality (Supplementary Table 2).

### Sensitivity analyses of the primary outcome

The sensitivity analysis showed that no significant alterations of the primary outcome were observed with the removal of one study, indicating that the results of pathological response in breast cancer patients who received nab-paclitaxel/sb-taxanes were relatively stable and reliable (Supplementary Figure S3). Selecting to use the random-effects or fixed-effects model for a meta-analysis based on the threshold of heterogeneity could be arbitrary. Thus, random-effects models were used to examine whether the results could be affected compared with the fixed-effects model. The results showed that the conclusion did not change (Supplementary Table S3).

### Quality of evidence

Table 3 shows the assessments of each outcome under the evaluation of GRADE method. In terms of pCR, a low certainty on the estimates was assessed, yet the results also presented a critical importance for the employment of nab-paclitaxel. ORR and OS both presented a serious risk of imprecision regarding the outcome. Besides from its important level of evidence, the evaluation for ORR also demonstrated a very low certainty as well. As for OS, the assessment showed a moderate level of certainty and an important level of importance. Finally, the evaluation for EFS presented a high certainty and an important level of importance. Therefore, the confidence in the observed alterations is relatively low, future studies are encouraged to update the recommendations of this study.

**Table 3:** GRADE evaluation of the studies

Certainty assessment							№ of patients		Effect		Certainty	Importance
№ of studies	Study design	Risk of bias	Inconsistency	Indirectness	Imprecision	Other considerations	nab-paclitaxel	sb-taxanes	Relative(95% CI)	Absolute(95% CI)		
pCR (assessed with: ypT0 ypN0)												
5	observational studies	not serious	not serious	not serious	not serious	none	429/1069 (40.1%)	333/1065 (31.3%)	**OR 1.52**(1.27 to 1.83)	**96 more per 1000**(from 54 more to 142 more)	⨁⨁◯◯LOW	CRITICAL
pCR (assessed with: ypT0/is ypN0)												
5	observational studies	not serious	not serious	not serious	not serious	none	379/1140 (33.2%)	315/1195 (26.4%)	**OR 1.40**(1.17 to 1.68)	**70 more per 1000**(from 32 more to 112 more)	⨁⨁◯◯LOW	CRITICAL
ORR												
4	observational studies	not serious	not serious	not serious	serious ^a^	none	832/1057 (78.7%)	848/1116 (76.0%)	**OR 1.19**(0.97 to 1.46)	**30 more per 1000**(from 6 fewer to 62 more)	⨁◯◯◯VERY LOW	IMPORTANT
OS												
2	randomised trials	not serious	not serious	not serious	serious ^a^	none	106/846 (12.5%)	130/819 (15.9%)	**OR 0.79**(0.60 to 1.04)	**29 fewer per 1000**(from 57 fewer to 5 more)	⨁⨁⨁◯MODERATE	IMPORTANT
EFS												
2	randomised trials	not serious	not serious	not serious	not serious	none	952 participants	949 participants	**HR 0.69**(0.57 to 0.85)[EFS]	**-- per 1000**(from -- to --)	⨁⨁⨁⨁HIGH	IMPORTANT
							–	0.0%		**-- per 1000**(from -- to --)		

*CI* Confidence interval; *OR* Odds ratio; *HR* Hazard Ratio

**Explanations**

^a^The CI includes the possibility of both harms or benefits

### Publication bias

Potential publication bias was assessed; there was no evidence of publication bias for the pooled analysis of pathological response and ORR (Supplementary Figure S4). It has to be noted that the assessment of publication bias is weak because of the small number of studies available for the funnel plot.

## Discussion

Our study is the first meta-analysis that evaluated the effects of neoadjuvant nab-paclitaxel versus sb-taxanes for breast cancer and reported both short-term and long-term outcomes. The results showed that neoadjuvant nab-paclitaxel is effective and with reasonable toxicities.

The studies included in the present study analyzed patients with demographic and clinical characteristics similar to those of the studies included in the previous meta-analysis of neoadjuvant nab-paclitaxel [14]. Furthermore, the chemotherapies used with nab- or sb-paclitaxel were standard therapies supported by guidelines [2–4]. In the present meta-analysis, nab-paclitaxel was associated with a higher likelihood of achieving pCR compared with sb-taxanes, which is supported by the previous meta-analysis by Zong et al. [14]. The better efficacy observed for nab-paclitaxel might be due to the enhanced drug permeation and retention and to an increased local drug concentration at the tumor site [13, 32], which is achieved through receptor-mediated transcytosis [33]. Our results also showed that when using ypT0 ypN0 as pCR definition, the pooled proportion of patients with pCR (40.1% for nab-paclitaxel and 31.3% for sb-taxanes) was a little higher (33.2% for nab-paclitaxel and 26.4% for sb-taxanes) than when used ypT0/is ypN0 as pCR definition. This may due to not all used data in the two pCR definition arms that were from the same study. The variation in BC subtypes among different studies may contribute to this result. The trial by Gianni et al. (all patients with HER2-negative disease and 68% was HR-positive, weight 25.91%) used ypT0/is ypN0 as the definition for pCR outcome, whereas the trial by Moebus et al. (> 80% patients with TNBC or HER2-positive disease, weight 33.16%) used ypT0 ypN0 as the definition for pCR outcome. Indeed, we found that the pCR rates for nab-paclitaxel varied greatly across individual studies during our analysis, mainly attributed to the differences in patient and disease characteristics. Our subgroup analyses demonstrated that the pCR rates for nab-paclitaxel were higher in HER2-positive and TNBC patients and lower in HER2-negative, HR-positive patients, which is in line with the findings of Zong et al. [14]. Currently, the underlying mechanism for the modulation of response to taxanes by HR and HER2 in breast cancer is not entirely understood and requires further investigation [34]. However, higher pCR rates in the HER2-positive subtype may due to the use of HER2-targeted therapy, such as trastuzumab, etc. [35].

Paclitaxel every 2 weeks/weekly regimen is recommended by the National Comprehensive Cancer Network (NCCN) guideline for neoadjuvant treatment of TNBC, which is the most aggressive subtype of breast cancer [2]. Our results showed that neoadjuvant nab-paclitaxel had a higher pCR rate than neoadjuvant sb-taxanes when used weekly nab-paclitaxel in day 1, 8, 15 for four 3-week cycles/four two-weekly cycles regimen. This pCR rate for neoadjuvant nab-paclitaxel was also considerably higher than the overall pCR reported by a previous meta-analysis in TNBC patients treated with neoadjuvant chemotherapy [36]. In addition, our findings are consistent with the high response rates observed for nab-paclitaxel in the metastatic setting in TNBC [37, 38]. Despite a relatively low pCR rate for the HER2-negative, HR-positive breast cancer, pCR has been illustrated to be independently associated with patient prognosis regardless of cancer subtype [39]. A recent database study reported that neoadjuvant chemotherapy was more effective than neoadjuvant endocrine therapy in downstaging tumors in HER2-negative, estrogen receptor-positive locally advanced breast cancer [40]. Therefore, improved neoadjuvant chemotherapy efficacy might be especially beneficial in HER2-negative, HR-positive breast cancer. Our study found that nab-paclitaxel treatment was associated with a higher pCR rate than sb-taxanes for patients with HER2-negative, HR-positive disease. Regarding the proliferation index, patients with Ki-67 > 20% showed overall higher pCR rates than those with Ki-67 < 20%, as supported by a previous study [41]. Nab-paclitaxel showed better pCR rates in patients with Ki-67 > 20% compared with sb-taxanes, but not in patients with Ki-67 < 20%. Taken together, our data support the use of nab-paclitaxel as a reasonable treatment option for breast cancer in the neoadjuvant setting, especially in patients with HER2-negative HR-positive cancer and in high-risk patients (i.e., patients with TNBC and high Ki-67), but larger-scale confirmative studies are warranted.

pCR is a strong predictor for favorable long-term prognosis in breast cancer [42]. Nevertheless, it remains unclear how large a difference in pCR between nab-paclitaxel and sb-taxanes can translate into a difference in long-term clinical outcomes. With the recent availability of the 4-year outcome of the GeparSepto trial and the 5-year outcome of the ETNA trial, we could perform a pooled analysis of 1901 patients [21, 30]. The results showed that neoadjuvant nab-paclitaxel was associated with improved EFS compared with sb-taxanes. This improvement could be due, at least in part, to the fact that nanomedicine can better target cancer stem cells escaped from the primary tumor site, which are part of a subpopulation of tumor cells responsible for an important proportion of recurrences and metastases [43]. However, no statistically significant difference was observed for OS between the two treatments, though a trend of improved OS was noted for nab-paclitaxel. The lack of a more pronounced improvement in OS with nab-paclitaxel might be attributed to the availability of effective adjuvant therapy for early breast cancer and salvage regimens for metastatic breast cancer [2], which partially attenuated the effect of neoadjuvant treatments on OS. On the other hand, the power of our OS analysis was limited by the number of survival events. The overall low event number available for OS analysis at 4–5 years after neoadjuvant therapy further confirms the difficulty of incorporating OS as a primary endpoint in clinical trials for non-metastatic breast cancer.

In the present meta-analysis, there were no differences between nab-paclitaxel and sb-paclitaxel in most grade > 3 AEs. Nevertheless, the incidence of grade > 3 neuropathies was higher with nab-paclitaxel, most likely due to the higher dose of paclitaxel administered/delivered with nab-paclitaxel than with sb-paclitaxel. These results are similar to those of Zong et al. [14], who showed that there were no differences in the toxicity profiles of nab-paclitaxel and sb-paclitaxel in terms of severe AEs, but that neuropathies were increased with nab-paclitaxel. Notably, the occurrence of any-grade hypersensitivity events is decreased using nab-paclitaxel despite the use of premedication for sb-taxanes. Therefore, this allows reductions in health care costs [44, 45]. Nevertheless, the meta-analysis of the safety data must be taken with caution as the number of studies that could be included was small, and heterogeneity was high.

This study has limitations. Not all the included studies were RCTs, leading to potential bias. The included studies were conducted at various institutions and in various countries and may have a potential bias in local cancer management policies and in reporting the types of adverse events. Importantly, different doses of nab-paclitaxel and comparator regimens were used in the included studies. In addition, different definitions of ORR were also used: 1) the proportion of patients who attain either a CR or a PR during the study, evaluated according to the RECIST criteria (v 1.1) purposely modified for this protocol and detailed in the SAP [16]; 2) using the Response Evaluation Criteria In Solid Tumors, version 1.0 [17]; 3) clinical response in breast and nodes was assessed after taxane treatment and before surgery and was defined by the modified WHO criteria [20]; or 4) the clinical tumor response was as a CR if there was no clinical evidence of palpable tumors in either the breast or axilla at the time of surgery, while a reduction of total tumor size by > 30% at the time of surgery was considered a PR [15].

Regarding survival, the EFS and OS results should be taken with caution because only two studies reported long term outcomes. Likewise, AEs results should be explained cautiously, due to some analyses of AEs were driven by only one study. Nevertheless, in the original study review process prior to data analysis, this study was eligible based on the inclusion/exclusion criteria, and, methodologically, it could not be excluded after the analysis. In addition, among the non-randomized studies, Huang et al. [15] reported the unadjusted ORs, while Xie et al. [18] did not report any OR. Therefore, the observational studies carry a risk of selection bias, indication bias, and immortal time bias, but those biases could not be controlled in the present meta-analysis. Only studies published in English were included, which is a bias. Publication bias is a major concern in all forms of meta-analysis, and it is likely that unpublished negative results were not included. The quality evaluation is also a concern, and quality evaluation is crude and somewhat subjective.

## Conclusion

In conclusion, this meta-analysis suggests that compared with conventional sb-taxanes, neoadjuvant nab-paclitaxel could improve pCR rate and EFS in non-metastatic breast cancer and could be especially beneficial in the HER2-negative HR-positive and high-risk disease types. In addition, the substitution of sb-taxanes with nab-paclitaxel is associated with reasonable toxicities.

## Supplementary Information

**Additional file 1: Supplementary Figure S1.** Forest plot of event-free survival (A) and overall survival (B) for neoadjuvant nab-paclitaxel versus sb-paclitaxel in the treatment of breast cancer. Grey boxes demonstrate the effect size of each study, and their size is proportional to the weight given to each study. The whiskers bilateral to each grey box represent the 95% confidence interval (CI) of each study’s effect size. A fixed model was used for hazard ratio (HR) or odds ratio (OR) calculation. Abbreviations: nab-P, nanoparticle albumin-bound paclitaxel; sb-T, solvent-based taxanes.

**Additional file 2: Supplementary Figure S2.** Methodological quality summary: review authors’ judgments about each methodological quality item for included randomized controlled trials according to the Cochrane risk bias tool.

**Additional file 3: Supplementary Figure S3.** Sensitivity analysis of the primary outcome.

**Additional file 4: Supplementary Figure S4.** (A) Funnel plot of pathological complete response (pCR). (B) Funnel plot of objective response rate (ORR).

**Additional file 5.**

## Data Availability

The datasets used and/or analyzed during the current study are available from the corresponding author on reasonable request.

## Abbreviation

RCTs: Randomized controlled trials
AEs: Adverse events
pCR: pathological complete response
FDA: Food and Drug Administration
EMA: European Medicines Agency
sb: solvent-based
DFS: Disease-free survival
OS: Overall survival
ASCO: American Society of Clinical Oncology
ESMO: European Society of Medical Oncology Conference
SABCS: San Antonio Breast Cancer Symposium
NOS: Newcastle-Ottawa scale
OR: Odds ratio
HR: Hazard ratio
CIs: Confidence intervals
