# Supplementary figures and images for: Comparison between nab-paclitaxel and solvent-based taxanes as neoadjuvant therapy in breast cancer: a systematic review and meta-analysis
Source: BMC Cancer. 2021 Feb 4;21:118. doi: 10.1186/s12885-021-07831-7 (PMC7863369; doi:10.1186/s12885-021-07831-7)

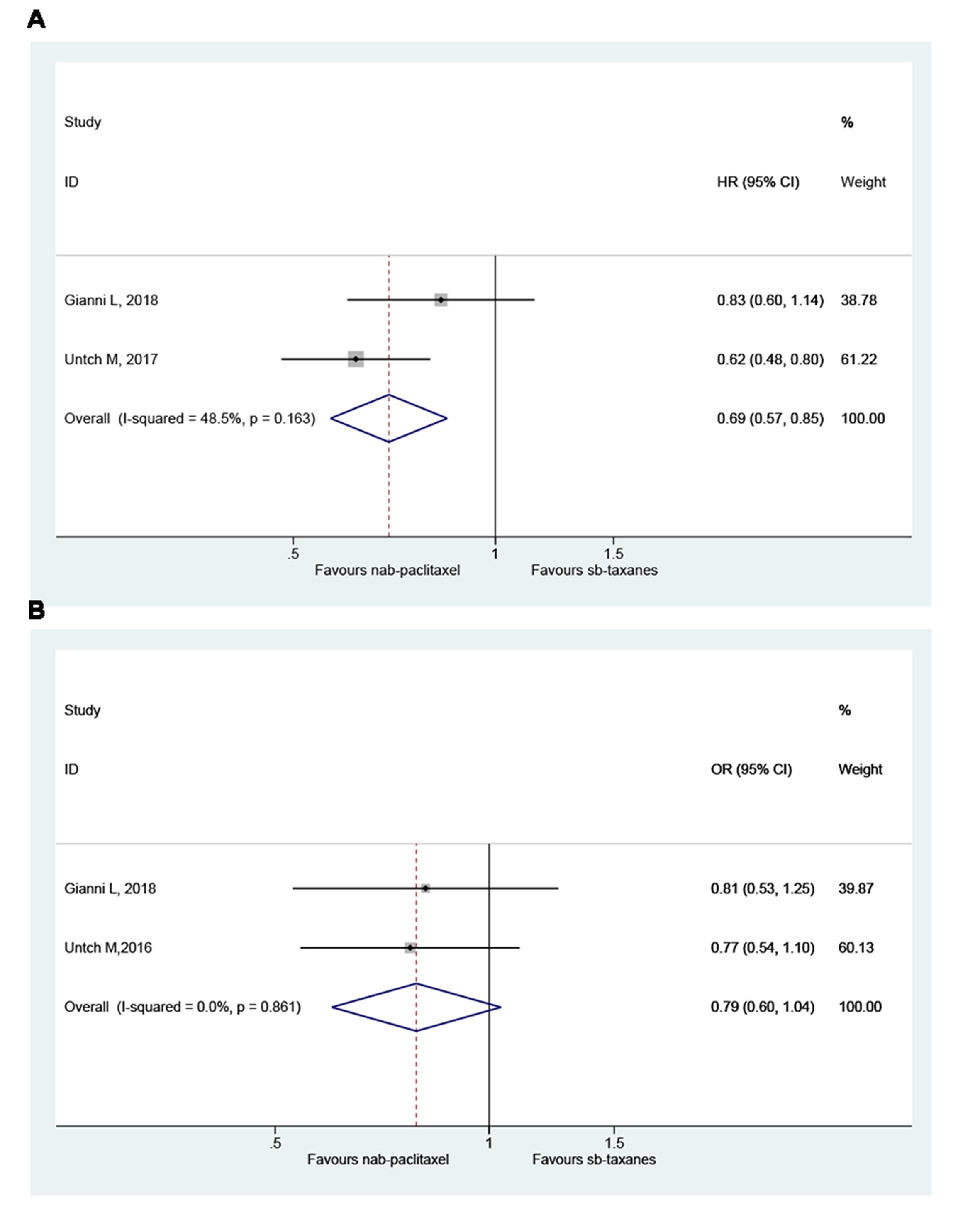

Supplement: Supplementary file 1 — Additional file 1: Supplementary Figure S1. Forest plot of event-free survival (A) and overall survival (B) for neoadjuvant nab-paclitaxel versus sb-paclitaxel in the treatment of breast cancer. Grey boxes demonstrate the effect size of each study, and their size is proportional to the weight given to each study. The whiskers bilateral to each grey box represent the 95% confidence interval (CI) of each study’s effect size. A fixed model was used for hazard ratio (HR) or odds ratio (OR) calculation. Abbreviations: nab-P, nanoparticle albumin-bound paclitaxel; sb-T, solvent-based taxanes. [file 12885_2021_7831_MOESM1_ESM.tif]

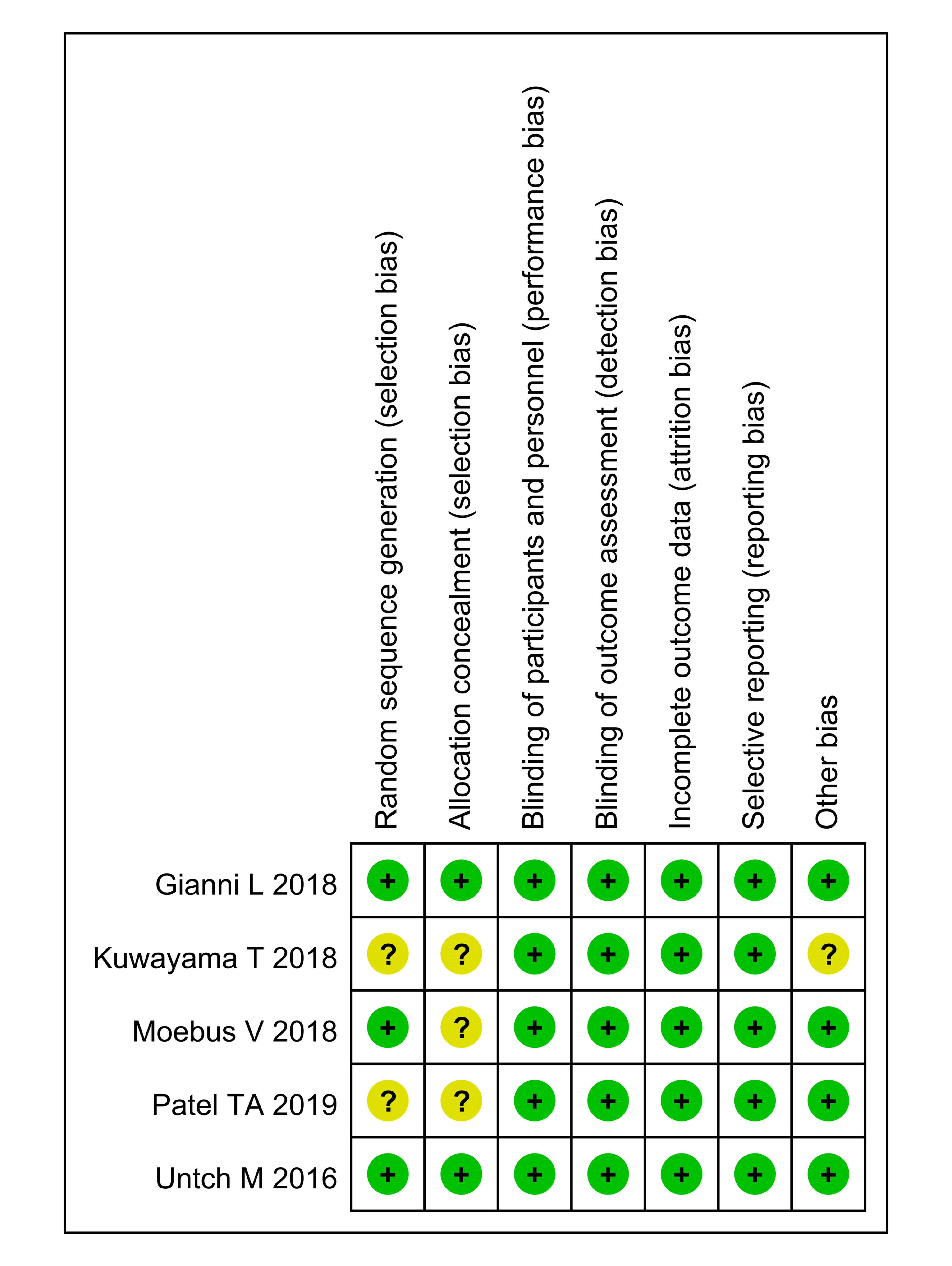

Supplement: Supplementary file 2 — Additional file 2: Supplementary Figure S2. Methodological quality summary: review authors’ judgments about each methodological quality item for included randomized controlled trials according to the Cochrane risk bias tool. [file 12885_2021_7831_MOESM2_ESM.tif]

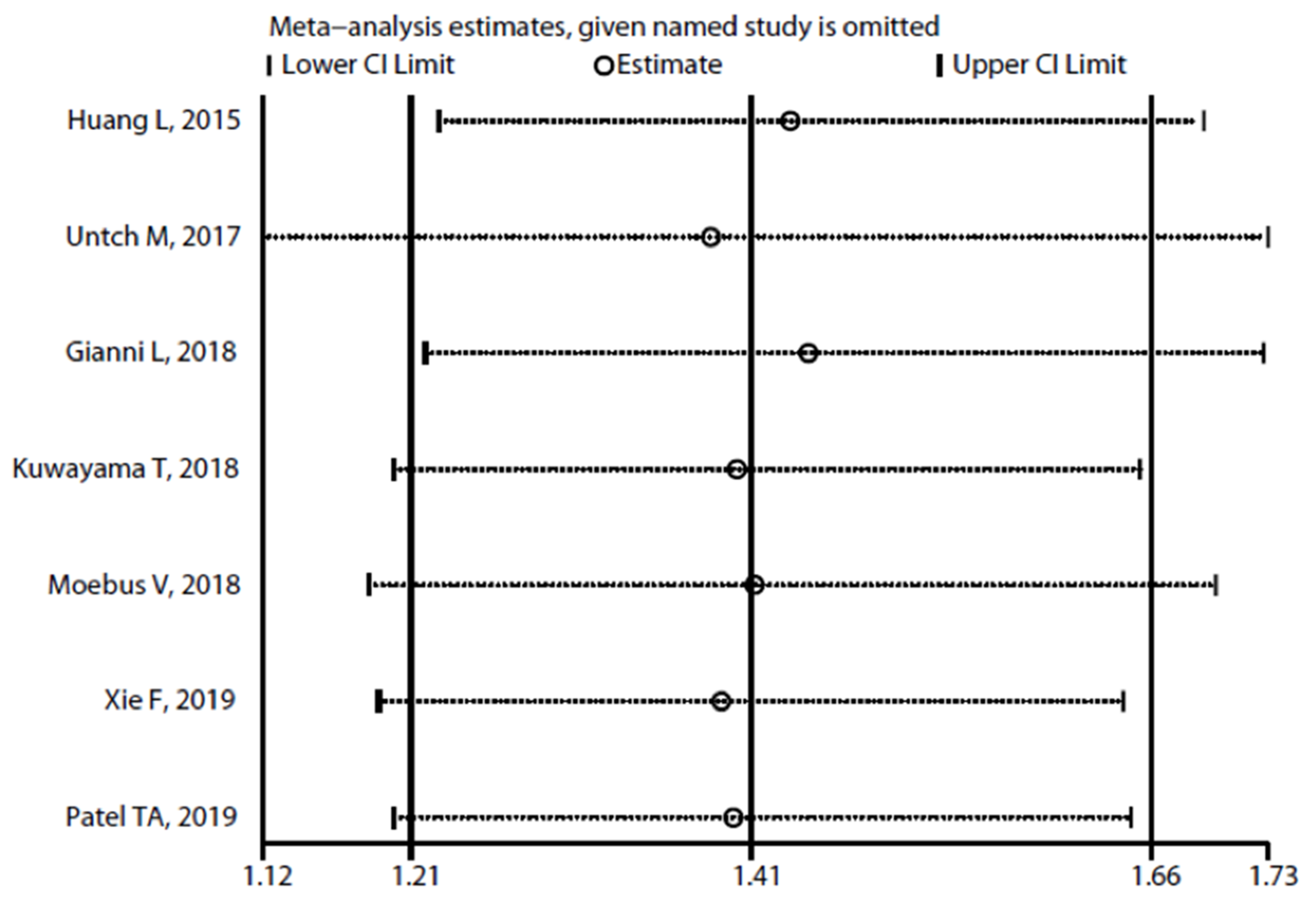

Supplement: Supplementary file 3 — Additional file 3: Supplementary Figure S3. Sensitivity analysis of the primary outcome. [file 12885_2021_7831_MOESM3_ESM.tif]

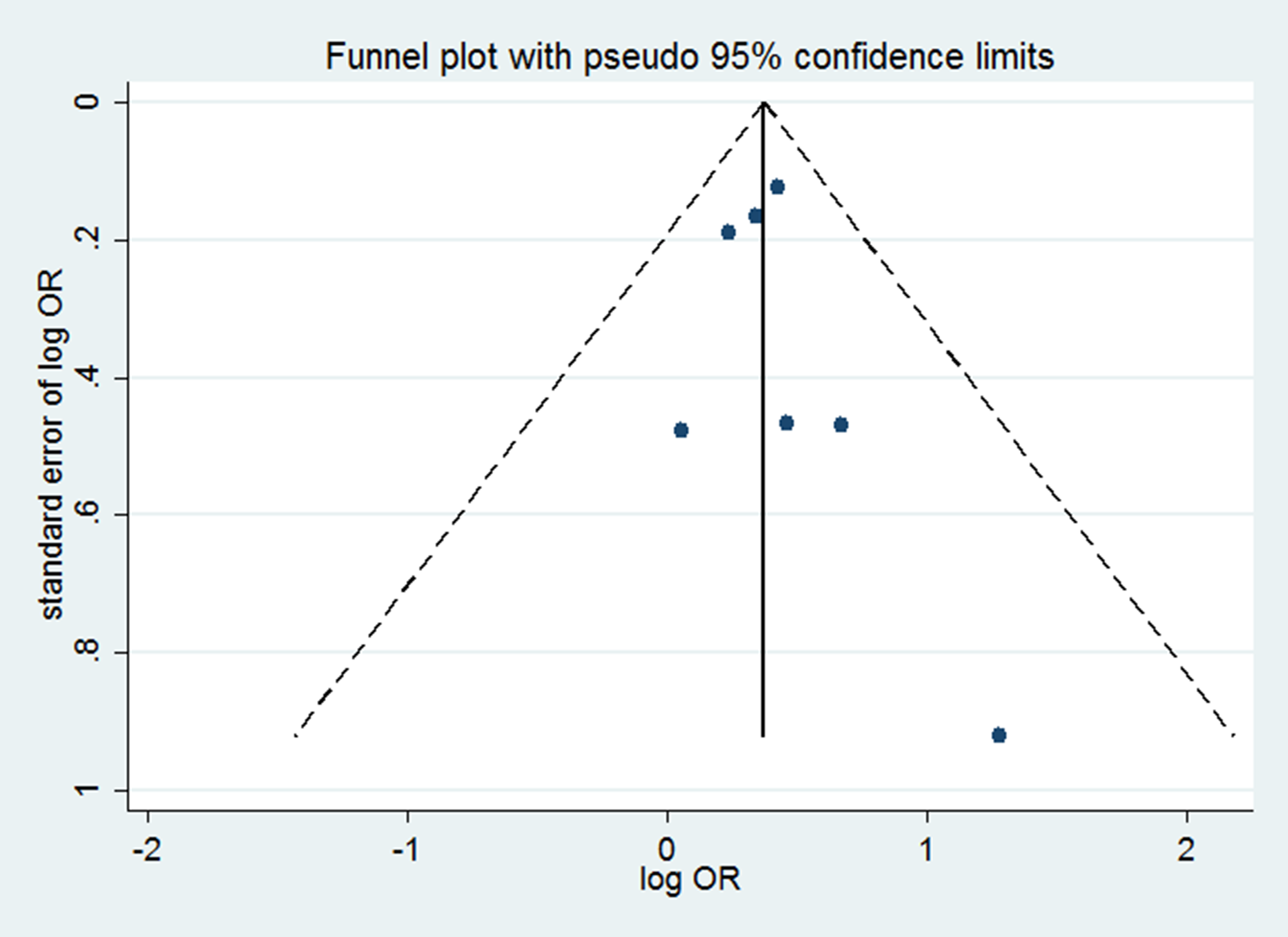

Supplement: Supplementary file 4 — Additional file 4: Supplementary Figure S4. (A) Funnel plot of pathological complete response (pCR). (B) Funnel plot of objective response rate (ORR). [file 12885_2021_7831_MOESM4_ESM.zip › Figure S4AR4.tif]

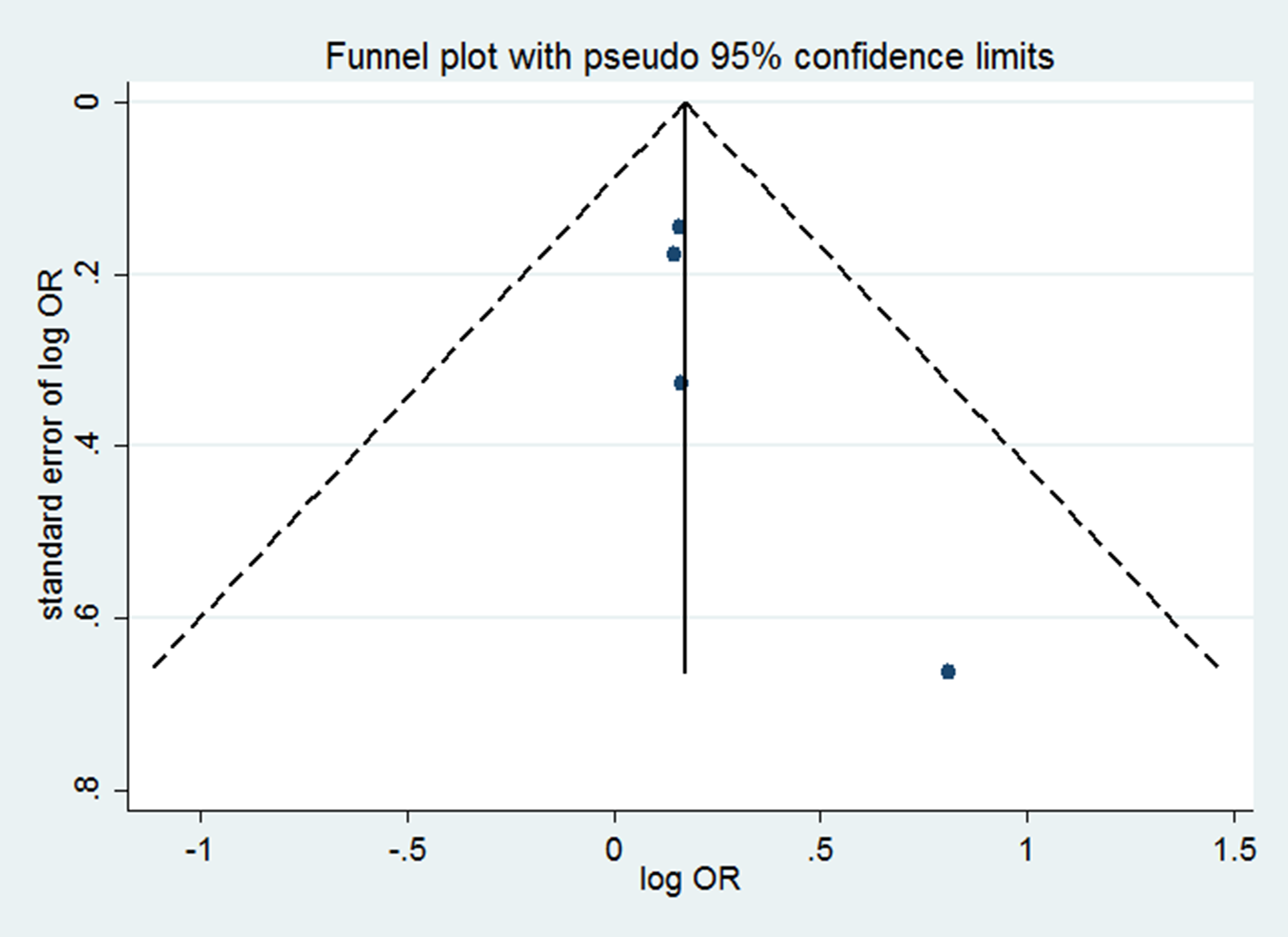

Supplement: Supplementary file 4 — Additional file 4: Supplementary Figure S4. (A) Funnel plot of pathological complete response (pCR). (B) Funnel plot of objective response rate (ORR). [file 12885_2021_7831_MOESM4_ESM.zip › Figure S4BR4.tif]
