# Supplementary material for: Comparison between nab-paclitaxel and solvent-based taxanes as neoadjuvant therapy in breast cancer: a systematic review and meta-analysis
Source: BMC Cancer. 2021 Feb 4;21:118. doi: 10.1186/s12885-021-07831-7 (PMC7863369; doi:10.1186/s12885-021-07831-7)
Supplement: Supplementary file 5 — Additional file 5. [file 12885_2021_7831_MOESM5_ESM.docx]

**Supplementary Table 1. Search strategies**

| Search strategy | |
| --- | --- |
| PubMed |  |
| #1 | (((("Breast Neoplasms"[Mesh]) OR (((((((((((((((((((((((((((((((((((((Breast Neoplasm[Title/Abstract]) OR Neoplasm, Breast[Title/Abstract]) OR Breast Tumors[Title/Abstract]) OR Breast Tumor[Title/Abstract]) OR Tumor, Breast[Title/Abstract]) OR Tumors, Breast[Title/Abstract]) OR Neoplasms, Breast[Title/Abstract]) OR Breast Cancer[Title/Abstract]) OR Cancer, Breast[Title/Abstract]) OR Mammary Cancer[Title/Abstract]) OR Cancer, Mammary[Title/Abstract]) OR Cancers, Mammary[Title/Abstract]) OR Mammary Cancers[Title/Abstract]) OR Malignant Neoplasm of Breast[Title/Abstract]) OR Breast Malignant Neoplasm[Title/Abstract]) OR Breast Malignant Neoplasms[Title/Abstract]) OR Malignant Tumor of Breast[Title/Abstract]) OR Breast Malignant Tumor[Title/Abstract]) OR Breast Malignant Tumors[Title/Abstract]) OR Cancer of Breast[Title/Abstract]) OR Cancer of the Breast[Title/Abstract]) OR Mammary Carcinoma, Human[Title/Abstract]) OR Carcinoma, Human Mammary[Title/Abstract]) OR Carcinomas, Human Mammary[Title/Abstract]) OR Human Mammary Carcinomas[Title/Abstract]) OR Mammary Carcinomas, Human[Title/Abstract]) OR Human Mammary Carcinoma[Title/Abstract]) OR Mammary Neoplasms, Human[Title/Abstract]) OR Human Mammary Neoplasm[Title/Abstract]) OR Human Mammary Neoplasms[Title/Abstract]) OR Neoplasm, Human Mammary[Title/Abstract]) OR Neoplasms, Human Mammary[Title/Abstract]) OR Mammary Neoplasm, Human[Title/Abstract]) OR Breast Carcinoma[Title/Abstract]) OR Breast Carcinomas[Title/Abstract]) OR Carcinoma, Breast[Title/Abstract]) OR Carcinomas, Breast[Title/Abstract]))) |
| #2 | ("Neoadjuvant Therapy"[Mesh]) OR (((((((((Neoadjuvant Therapies[Title/Abstract]) OR Therapies, Neoadjuvant[Title/Abstract]) OR Therapy, Neoadjuvant[Title/Abstract]) OR Neoadjuvant Treatment[Title/Abstract]) OR Neoadjuvant Treatments[Title/Abstract]) OR Treatment, Neoadjuvant[Title/Abstract]) OR Treatments, Neoadjuvant[Title/Abstract]) OR neoadjuvant chemotherapy[Title/Abstract]) OR preoperative chemotherapy[Title/Abstract]) |
| #3 | ("Albumin-Bound Paclitaxel"[Mesh]) OR ((((((((((((((Albumin Bound Paclitaxel[Title/Abstract]) OR Paclitaxel, Albumin-Bound[Title/Abstract]) OR Protein-Bound Paclitaxel[Title/Abstract]) OR Paclitaxel, Protein-Bound[Title/Abstract]) OR Protein Bound Paclitaxel[Title/Abstract]) OR Abraxane[Title/Abstract]) OR ABI007[Title/Abstract]) OR ABI-007[Title/Abstract]) OR ABI 007[Title/Abstract]) OR nab-paclitaxel[Title/Abstract]) OR nanoparticle albumin-bound paclitaxel[Title/Abstract]) OR nanoparticle albumin bound paclitaxel[Title/Abstract]) OR 130-nm albumin-bound paclitaxel[Title/Abstract]) OR nab-PTX[Title/Abstract]) |
| #4 | #1 AND #2 AND #3 |
| Embase |  |
| #1 | 'breast cancer'/exp |
| #2 | 'Breast Neoplasm':ab,ti OR 'Neoplasm, Breast':ab,ti OR 'Breast Tumors':ab,ti OR 'Breast Tumor':ab,ti OR 'Tumor, Breast':ab,ti OR 'Tumors, Breast':ab,ti OR 'Neoplasms, Breast':ab,ti OR 'Breast Cancer':ab,ti OR 'Cancer, Breast':ab,ti OR 'Mammary Cancer':ab,ti OR 'Cancer, Mammary':ab,ti OR 'Cancers, Mammary':ab,ti OR 'Mammary Cancers':ab,ti OR 'Malignant Neoplasm of Breast':ab,ti OR 'Breast Malignant Neoplasm':ab,ti OR 'Breast Malignant Neoplasms':ab,ti OR 'Malignant Tumor of Breast':ab,ti OR 'Breast Malignant Tumor':ab,ti OR 'Breast Malignant Tumors':ab,ti OR 'Cancer of Breast':ab,ti OR 'Cancer of the Breast':ab,ti OR 'Mammary Carcinoma, Human':ab,ti OR 'Carcinoma, Human Mammary':ab,ti OR 'Carcinomas, Human Mammary':ab,ti OR 'Human Mammary Carcinomas':ab,ti OR 'Mammary Carcinomas, Human':ab,ti OR 'Human Mammary Carcinoma':ab,ti OR 'Mammary Neoplasms, Human':ab,ti OR 'Human Mammary Neoplasm':ab,ti OR 'Human Mammary Neoplasms':ab,ti OR 'Neoplasm, Human Mammary':ab,ti OR 'Neoplasms, Human Mammary':ab,ti OR 'Mammary Neoplasm, Human':ab,ti OR 'Breast Carcinoma':ab,ti OR 'Breast Carcinomas':ab,ti OR 'Carcinoma, Breast':ab,ti OR 'Carcinomas, Breast':ab,ti |
| #3 | #1 OR #2 |
| #4 | 'neoadjuvant chemotherapy'/exp |
| #5 | 'neoadjuvant therapy'/exp |
| #6 | 'preoperative chemotherapy'/exp |
| #7 | 'Neoadjuvant Therapies':ab,ti OR 'Therapies, Neoadjuvant':ab,ti OR 'Therapy, Neoadjuvant':ab,ti OR 'Neoadjuvant Treatment':ab,ti OR 'Neoadjuvant Treatments':ab,ti OR 'Treatment, Neoadjuvant':ab,ti OR 'Treatments, Neoadjuvant':ab,ti |
| #8 | #4 OR #5 OR #6 OR #7 |
| #9 | 'Albumin-Bound Paclitaxel' :ab,ti OR 'Albumin Bound Paclitaxel':ab,ti OR 'Paclitaxel, Albumin-Bound':ab,ti OR 'Protein-Bound Paclitaxel':ab,ti OR 'Paclitaxel, Protein-Bound':ab,ti OR 'Protein Bound Paclitaxel' :ab,ti OR 'Abraxane':ab,ti OR 'ABI007':ab,ti OR 'ABI-007':ab,ti OR 'ABI 007':ab,ti OR 'nab-paclitaxel':ab,ti OR 'nanoparticle albumin-bound paclitaxel':ab,ti OR 'nanoparticle albumin bound paclitaxel':ab,ti OR '130-nm albumin-bound paclitaxel':ab,ti OR 'nab-PTX':ab,ti |
| #10 | #3 AND #8 AND 9 |
| Cochrane |  |
| #1 | MeSH descriptor: [Breast Neoplasms] explode all trees |
| #2 | Breast Neoplasm:ti,ab,kw OR Neoplasm, Breast:ti,ab,kw OR Breast Tumors:ti,ab,kw OR Breast Tumor:ti,ab,kw OR Tumor, Breast:ti,ab,kw OR Tumors, Breast:ti,ab,kw OR Neoplasms, Breast:ti,ab,kw OR Breast Cancer:ti,ab,kw OR Cancer, Breast:ti,ab,kw OR Mammary Cancer:ti,ab,kw OR Cancer, Mammary:ti,ab,kw OR Cancers, Mammary:ti,ab,kw OR Mammary Cancers:ti,ab,kw OR Malignant Neoplasm of Breast:ti,ab,kw OR Breast Malignant Neoplasm:ti,ab,kw OR Breast Malignant Neoplasms:ti,ab,kw OR Malignant Tumor of Breast:ti,ab,kw OR Breast Malignant Tumor:ti,ab,kw OR Breast Malignant Tumors:ti,ab,kw OR Cancer of Breast:ti,ab,kw OR Cancer of the Breast:ti,ab,kw OR Mammary Carcinoma, Human:ti,ab,kw OR Carcinoma, Human Mammary:ti,ab,kw OR Carcinomas, Human Mammary:ti,ab,kw OR Human Mammary Carcinomas:ti,ab,kw OR Mammary Carcinomas, Human:ti,ab,kw OR Human Mammary Carcinoma:ti,ab,kw OR Mammary Neoplasms, Human:ti,ab,kw OR Human Mammary Neoplasm:ti,ab,kw OR Human Mammary Neoplasms:ti,ab,kw OR Neoplasm, Human Mammary:ti,ab,kw OR Neoplasms, Human Mammary:ti,ab,kw OR Mammary Neoplasm, Human:ti,ab,kw OR Breast Carcinoma:ti,ab,kw OR Breast Carcinomas:ti,ab,kw OR Carcinoma, Breast:ti,ab,kw OR Carcinomas, Breast:ti,ab,kw |
| #3 | #1 OR #2 |
| #4 | MeSH descriptor: [Neoadjuvant Therapy] explode all trees |
| #5 | Neoadjuvant Therapies:ti,ab,kw OR Therapies, Neoadjuvant:ti,ab,kw OR Therapy, Neoadjuvant:ti,ab,kw OR Neoadjuvant Treatment:ti,ab,kw OR Neoadjuvant Treatments:ti,ab,kw OR Treatment, Neoadjuvant:ti,ab,kw OR Treatments, Neoadjuvant:ti,ab,kw OR neoadjuvant chemotherapy:ti,ab,kw OR preoperative chemotherapy:ti,ab,kw |
| #6 | #4 OR #5 |
| #7 | MeSH descriptor: [Albumin-Bound Paclitaxel] explode all trees |
| #8 | Albumin Bound Paclitaxel:ti,ab,kw OR Paclitaxel, Albumin-Bound:ti,ab,kw OR Protein-Bound Paclitaxel:ti,ab,kw OR Paclitaxel, Protein-Bound:ti,ab,kw OR Protein Bound Paclitaxel:ab,ti,kw OR Abraxane:ab,ti,kw OR ABI007:ab,ti,kw OR ABI-007:ti,ab,kw OR ABI 007:ab,ti,kw OR nab-paclitaxel:ab,ti,kw OR nanoparticle albumin-bound paclitaxel:ab,ti,kw OR nanoparticle albumin bound paclitaxel:ab,ti,kw OR albumin-bound paclitaxel:ab,ti,kw OR nab-PTX:ab,ti,kw |
| #9 | #7 OR #8 |
| #10 | #3 OR #6 OR #9 |

**Supplementary Table 2.** Quality assessment of the observational studies included in the meta-analysis by the nine-star Newcastle–Ottawa Scale.

|  | Item | Huang 2015 [15] | Xie 2019 [18] |
| --- | --- | --- | --- |
| *SELECTION* | Representativeness of the Exposed Cohort | 1 | 1 |
|  | Selection of the Non-Exposed Cohort | 1 | 1 |
|  | Ascertainment of Exposure | 1 | 1 |
|  | Demonstration That Outcome of Interest Was Not Present at Start of Study | 1 | 1 |
| *COMPARABILITY* | Comparability of Cohorts on the Basis of the Design or Analysis | 2 | 2 |
| *OUTCOME* | Assessment of Outcome | 1 | 1 |
|  | Was Follow-Up Long Enough for Outcomes to Occur | 1 | 1 |
|  | Adequacy of Follow Up of Cohorts | 1 | 1 |
| Total |  | 9 | 9 |

Supplementary Table 3. Sensitive analysis of random-effect model.

|  |  | Fix-effect model | Random-effect model |
| --- | --- | --- | --- |
| ypT0 ypN0 | OR, 95% CI | 1.52 (1.27, 1.83) | 1.52 (1.26, 1.83) |
| ypT0/is ypN0 | OR, 95% CI | 1.40 (1.17, 1.68) | 1.40 (1.16, 1.66) |
| RCT | OR, 95% CI | 1.45 (1.23, 1.72) | 1.45 (1.23, 1.72) |
| Non-RCT | OR, 95% CI | 1.46 (0.77, 2.78) | 1.45 (1.23, 1.71) |
| paclitaxel | OR, 95% CI | 1.46 (1.21, 1.77) | 1.46 (1.21, 1.77) |
| docetaxel | OR, 95% CI | 1.43 (1.05, 1.94) | 1.43 (1.05, 1.94) |
| HER2- | OR, 95% CI | 1.60 (1.26, 2.03) | 1.61 (1.19, 2.18) |
| HER2-, HR+ | OR, 95% CI | 1.53 (1.07, 2.19) | 1.50 (1.05, 2.16) |
| TNBC | OR, 95% CI | - | 1.86 (0.99, 3.51)* |
| HER2+ | OR, 95% CI | 1.37 (0.95, 2.00) | 1.36 (0.83, 2.21) |
| HER2+HR- | OR, 95% CI | - | 0.90 (0.26, 3.19)* |
| HER2+HR+ | OR, 95% CI | 1.45 (0.93, 2.26) | 1.93 (0.66, 5.60) |
| Weekly | OR, 95% CI | 1.17 (0.71, 1.91) | 1.17 (0.71, 1.91)* |
| Every 2 weeks | OR, 95% CI | 2.82 (1.74, 4.58) | 2.95 (1.54,5.67)* |
| ki67 >20% | OR, 95% CI | 1.63 (1.26, 2.12) | 1.63 (1.26, 2.12) |
| ki67 <20% | OR, 95% CI | 1.41 (0.87, 2.27) | 1.41 (0.87, 2.28) |
| ORR | OR, 95% CI | 1.19 (0.97, 1.46) | 1.19 (0.97, 1.46) |
| EFS | HR, 95% CI | 0.69 (0.57, 0.85) | 0.71 (0.53, 0.94) |
| OS | OR, 95% CI | 0.79 (0.60, 1.04) | 0.79 (0.60, 1.03) |

The random effects model was used in the original analyses when Cochran's Q test P<0.10 or I2>50%.
